# Notes and News

**Published:** 1895-03-23

**Authors:** 


### Notes and News.

Collected and Communicated.

HOSPITAL MEETINGS.

University College Hospital.—Annual General Meeting in the Board Room, Friday, March 22nd, at 4 p.m.

National Hospital for Paralysed and Epileptic.—Biennial Festival Dinner, Tuesday 22nd, Whitehall Rooms, Hotel Metropole.

Orphan Working School.—Festival dinner at Whitehall Rooms, Hotel Metropole, May 2nd.

Poplar Hospital.—Annual General Meeting in the Outpatient Waiting Room on Tuesday, March 26th, at 4.30.

Royal London Ophthalmic Hospital.—Annual General Meeting in the Board Room of the Hospital, on Tuesday, March 26th, at 12 noon.

Royal Hospital for Incurables.—Annual Dinner on Thursday, May 23rd, at the Albion Tavern, Aldersgate Street. Earl Compton will preside.

H.R.H. the Duchess of York has become a patroness of the Chelsea Hospital for Women.

The number of hospital beds available in New York is now 5,272. Ten years ago less than half this number existed.

The complimentary dinner to Sir John Erichsen has been postponed until March 27th, owing to the recent illness of that eminent surgeon.

The appeal made for St. Thomas's Hospital is still being generously responded to, we are glad to learn. Messrs. Nobles and Hoare have sent a contribution of £250.

Through the generosity of Mr. Frederick Webb, of Maida Vale, an annual prize in bacteriology is to be founded at St. George's Hospital, he having presented the hospital with £1,000 for the purpose.

The adoption of a common language for medicine does not seem to be an event of the immediate future. At the Congress of Hygiene and Demography to be held at Madrid this autumn, six languages are to be permissible for the discourses.

The new Waynflete Professor of Physiology at Oxford is Mr. Francis Gotch, who vacates the chair of Physiology at University College, Liverpool, for his present post. Professor Gotch's principal contributions to physiology were made during his Oxford residence. He has held several important posts during his brilliant career.

The presidency of the annual meeting of the British Association this year in Liverpool is to be held by Sir Joseph Lister.

The new Fever Hospital at Birkenhead was opened recently by the Mayor. The hospital has accommodation for 44 beds, there being plenty of room on the site to increase this number if necessary.

Not long ago a toiler in the paths of fiction turned out a humorous little sketch in which he caused a number of plain and elderly females to cross the ocean in pursuit of beauty offered to them by a professed discoverer of a "beautiful for ever" philtre. But truth is little behind fiction. We are informed that a "beauty specialist" really exists in the flesh, domiciled in New York. This modern magician is not a Madame Rachel. Her operations are more than skin deep. She aims at the transformation of the offending feature, or the whole physiognomy if need be. So far we have met no patient, but were the specialist to establish a hospital we doubt not that funds would be abundant and "empty beds" unknown.

The committee of the Miller Hospital, Greenwich, owing to the steadily increasing number of applicants for relief, find that it is absolutely imperative to make an urgent appeal for funds at once for the provision of further accommodation. The attendances of outpatients during the past year were upwards of 50,000. It has become especially necessary that provision be made for the reception of the large number of accidents that are continually being brought to the doors of the hospital. In 1885 there were 47; in 1894 close upon 4,000, entailing some 18,000 dressings. The committee, therefore, in issuing this appeal, have in view the following scheme: (1) The building of a new operation room; (2) the improvement and enlargement of the out-patient department; (3) the extension of the ward accommodation of the hospital. It is estimated that a sum of about £10,000 will be necessary for this purpose. Her Majesty the Queen has been graciously pleased to contribute the sum of £25 to the fund. Subscriptions will be thankfully received by the Secretary, at the hospital, Greenwich Road, or at any branch of the London and County Bank.

The National Hospital for the Paralysed and Epileptic is making a special appeal for funds on the occasion of their biennial festival which is to take place in April. The special objects of the appeal are (1) the maintenance of the hospital during the next two years; (2) the erection of a new convalescent home; (3) the foundation of pensions for the incurable. The appeal is a strong one, and will be most generally sympathised with. The undoubted excellence of the institution is beyond dispute; the advantages of a convalescent home for such a work, where convalescence is so protracted, cannot be denied; and the especial suitability and necessity of pensions for sufferers from paralysis and epilepsy are most obvious. In regard to the convalescent home, no institution of the kind will receive epileptic patients, and therefore a home under the supervision and the excellent management of the National Hospital is extremely desirable. It is proposed to place the new home on a good site at East Finchley, and the sum estimated as necessary for the purpose is £4,000. We trust that Mr. Rawlings will be able to announce the greater part of this sum collected at the forthcoming festival.

